# The Daily Basic Psychological Need Satisfaction and Work Engagement of Nurses: A ‘Shortitudinal’ Diary Study

**DOI:** 10.3390/healthcare10050863

**Published:** 2022-05-06

**Authors:** Jo-Mari Liebenberg, Salomé E. Scholtz, Leon T. De Beer

**Affiliations:** WorkWell Research Unit, Faculty of Economic and Management Sciences, North-West University, Potchefstroom 2531, South Africa; jomarilieb@gmail.com (J.-M.L.); salomescholtz01@gmail.com (S.E.S.)

**Keywords:** need satisfaction, nursing, public healthcare, work engagement, self-determination theory, basic need satisfaction, psychological need satisfaction

## Abstract

Nurses’ satisfaction and work engagement have been linked to patient outcomes. Nightshift nurses provide healthcare to the population and experience unique challenges in performing their healthcare tasks. Therefore, the current study aimed to investigate the daily basic needs satisfaction and work engagement of nightshift nurses in accordance with the satisfaction of three basic psychological needs as indicated by the Self-Determination Theory. A quantitative, ‘shortitudinal’ design (diary study; over a few days) with a multi-level research approach using a daily diary survey method was completed by a convenience sample of nurses working the nightshift in a public hospital (*n* = 33). The results revealed that the daily need for autonomy and need for relatedness did not significantly predict variance in daily work engagement. However, need for competence did significantly predict variance in daily work engagement, and general emotional load explained significant variability in daily need satisfaction of competence. Lastly, general role clarity had a negative impact on the daily variability in work engagement. This study provides healthcare organisations with explanations for variance in nursing performance and suggests possible interventions to address nursing outcomes in accordance with the three basic needs of nightshift nurses in daily activity.

## 1. Introduction

Nurses are pivotal in promoting the sustainability and efficiency of the healthcare system [1], especially in the current global ageing context where more pressure is placed on the healthcare system for more extended chronic care [2,3]. Providing 24/7 patient care across the globe is only possible due to nurses; however, mismanaged nurse shift work can often lead to various diseases, fatigue and low levels of alertness and performance [4]. A study on nurses in long-term care units found an increase in turnover within two years of employment for nurses who worked nightshifts, which caused severe sleep disruption [5]. Moreover, nightshift nurses experience lower staffing levels compared to dayshifts, reduced access to expert advice and increased responsibility due to the lack of supervisory presence, staffed physicians or other specialists in the hospital [6]. In developing countries, nurse-to-patient ratios are also generally low and create negative experiences for nurses, most evident by the recent COVID-19 pandemic [7]. This situation requires nurses to rely on their judgement, experience and knowledge when making critical patient care decisions. Nurses working with limited resources face additional barriers to delivering expected patient-centred care [8], which lowers their emotional wellbeing [9].

Considering the challenges faced by nurses, Keyko et al. [10] suggest researchers focus on work engagement in nursing practice. Based on the self-determination theory (SDT), a theory on environmental factors’ influences on human motivation, the impact of varied environmental factors and job characteristics such as job design, managerial styles, and pay contingencies impact nurses’ motivation, which is greatly mediated by their basic psychological needs [11]. If the basic needs for autonomy, competence and relatedness of nurses are supported within their work environment, it enables them to thrive, feel motivated and be engaged at work [12]. Positive work engagement is therefore valuable to the performance of nurses and organisational outcomes [10].

Currently, the healthcare outcomes of countries such as South Africa is concerning, with underperformance, poor healthcare [13], high rates of accidents, and nurse turnover [14]. Nurses are the largest part of the South African healthcare workforce [15] and are faced with a unique case study; the healthcare system has been shaped by powerful historical and social forces (namely Apartheid) [16], and is considered the epicentre of the Human Immunodeficiency Virus (HIV) and acquired immunodeficiency syndrome (AIDS) epidemic [17]. Currently, the country is in a nursing crisis due to a lack of interest in the nursing profession, low caring ethos, unsupportive human resources and a mismatch between community and carer needs [3,18]. The situation is further exasperated by South Africa’s aim to provide free healthcare, leading to an ever-expanding scope of nurse education and responsibility, where nurses take up more healthcare duties to provide health services without the needed resources and staff [3]. Nurses, especially within the public sector, report a lack of support and skills shortages, which lower healthcare quality [19].

According to Michel et al. [20], 50% of the country’s total health expenditure is spent on 16% of the population funded by private medical insurance or people who personally pay for their medical services. In contrast, the public health sector, with its insufficient and mismanaged resources, is controlled by the South African Department of Health, which provides free healthcare to approximately 84% of the population who lack the ability to pay for healthcare services or private medical schemes [20]. Nurses providing healthcare to South Africans are also severely outnumbered. The country’s total registered nursing workforce, comprising registered, enrolled, and auxiliaries, was estimated at 218 people per nurse [21]. For example, the North West province had an estimated 17,851 nurses for a population of 4,122,854 people [21]. Overall, South African nurses are dissatisfied with their employment citing limited available workspaces, safety, material and human resources, quality care [9], occurrences of back pain [22], obesity [23], patient loads and shift work [1].

Despite an increase in research on the effects of nurse shift work in a variety of settings [4], research gaps remain as previous research mainly collected data using cross-sectional surveys, that are outdated, sampled from both private and public sectors and did not consider work engagement concerning need satisfaction for South African public sector night nurses specifically [1,24,25,26,27,28,29]. High-quality healthcare is the ultimate goal in healthcare systems worldwide [30], and context-specific research is imperative in formulating suitable strategies for improving nurse retention and healthcare overall, as experiences can be influenced by context, culture and geographical regions [31].

Therefore, the current study aimed to investigate nurses’ daily basic needs satisfaction and work engagement working nightshifts in a public hospital. This study also implemented a quantitative, ‘shortitudinal’ design (diary study; longitudinal over seven days) and a multi-level research approach, which has more than one measurement point and allows for demonstrating both between-person and within-person variations of need satisfaction and work engagement among nurses daily [31,32].

### 1.1. Self-Determination Theory

The SDT is a universal theory of human motivation that has been used to effectively predict human behaviour [33] in various fields such as healthcare and management [34] for more than 40 years [35]. The theory describes the impact of social environments on the motivation, behaviour and wellbeing of employees by focusing on fundamental aspects of personality development, self-regulation and basic psychological needs [36]. Therefore, the SDT is well suited to address motivation and engagement in the workplace [37] based on three psychological needs: autonomy, relatedness and competence [38]. The SDT is driven by intrinsic motivation, which determines people’s thoughts, feelings and behaviour [34,38]. The SDT reflects on the skills, knowledge and beliefs a person might use and act on, especially in an environment where reaching goals and desired outcomes is valued [39]. Deci et al. [11] suggested that organisations must consider the self-determination theory concepts when creating policies, practices and environments that promote both wellness and high-quality performance of employees. Based on their suggestion, this study will consider the SDT to assist healthcare organisations in identifying aspects that will enable the wellbeing and work engagement of nurses. The SDT consists of five mini-theories: cognitive evaluation theory, organismic integration theory, causality orientations theory, basic needs theory, and goal content theory [40,41]. This study will focus on the fourth mini-theory, namely the basic needs theory.

### 1.2. Basic Psychological Need Satisfaction Theory

Van den Broeck et al. [42] explain that the basic needs satisfaction theory reveals that all people are optimally motivated and experience wellbeing regardless of their gender, social class and cultural context if their basic needs for autonomy, competence and relatedness are satisfied. The basic needs are ‘universal nutrients’ necessary for optimal functioning and beneficial for every human being [36]. The need for autonomy refers to a need for acting independently, having the full willingness and experiencing a choice when acting [42]. Reis et al. [43] regard the need for autonomy as essential for individuals’ wellbeing, and previous research shows patient outcomes improve with high nurse autonomy. However, autonomy within the nursing profession refers to nurses having the competence to apply their clinical and organisational knowledge within a context where nurse roles, behaviours and responsibilities are clearly defined [44].

The need for competence refers to the desire to experience effectiveness during the mastering of a task, or to bring about the desired effect through behaviour [43]. South African nurses’ competence is measured by their ability to adapt and integrate; professional judgement, attitudes and values, clinical skills, knowledge, leadership, communication, organisational and critical thinking in different circumstances [45]. As the most significant part of the healthcare workforce, their competence is considered fundamental to improving the quality of the free healthcare system in South Africa [46]. This highlights the importance, in this study, of considering nurses’ need for competence and the effects thereof on their daily work engagement. Furthermore, the need for relatedness refers to the mutual feeling of having supportive connections with colleagues and having a sense of connectedness with others [33], where people internalise and accept the values of those around them [47]. Thus, according to the SDT, if healthcare organisations create a work environment that supports these three psychological needs, nurses can thrive in the workplace, experience job satisfaction and function optimally [11,48], which consequently improve patient outcomes [10,31]. In line with this suggestion, this study focused on determining the dynamics of the need for autonomy, competence, and relatedness, respectively, with nurses’ work engagement within a multi-level data context.

### 1.3. Job Demands–Resources Model

The Job Demands–Resources model (JD-R model) is regarded as exceptionally apt in helping organisations and practitioners identify factors that influence employee functioning and wellbeing [49]. The theory’s core suggests that all occupations have job characteristics that can be divided into two different categories, namely job demands and job resources [50].

Job demands refer to the physical, social, or organisational aspects of a job that require continuous physical or mental skills or effort [51]. Job resources refer to the physical, social, psychological, or organisational aspects of a job required to achieve work goals and assist employees in dealing with job demands [52]. In other words, job resources help employees to achieve work goals, reduce job demands and/or stimulate personal growth [50]. Bakker [52] points out that job resources have been identified as the main drivers that predict and contribute to work engagement over time and on a daily basis. Bakker [52] states that engaged employees should be exposed to an abundance of job resources that they can use and invest in their work. The more job resources available to employees, the better the employees are able to cope with their daily job demands [53].

The JD-R model describes how job resources and job demands influence work engagement and burnout. This triggers two relatively independent processes: health impairment (employees become exhausted due to an imbalance between job demands and job resources) and motivational processes (due to sufficient resources, employees are engaged in performing their job) [12]. The model proposes that high job demands lead to the health impairment process, and increased job resources lead to the motivational process [54]. In other words, the JD-R model suggests that high job demands relate to burnout and high job resources to engagement [55]. Despite the study by Hontake and Ariyoshi [56] on work engagement among nurses in Japan not being able to adequately clarify the relationship between job demands, job resources and work engagement, research shows that work engagement increases when employees are confronted with job demands while simultaneously having access to sufficient job resources [52]. This is supported by the earlier multi-level study conducted by Vera et al. [57] in which it was found that the greater the availability of job resources at the individual level (i.e., job autonomy) and at the team level (i.e., colleague and supervisor social support), the more likely it is that nurses will feel engaged.

There are various authors that argue that the JD-R model can be applied to a variety of work environments and enables researchers to obtain a quick idea of what can be expected in a particular situation and what concepts should be targeted to improve the health, wellbeing and performance of the workforce [53,54]. Keyko et al. [10] concluded that the JD-R model offers clear and valuable insight for practice and should, therefore, be used when focusing on the work engagement of nurses. Thus, the current study refers to the JD-R model but focuses more explicitly on the job resources and engagement aspects of the motivational process for nurses in the public sector at the general level.

### 1.4. Work Engagement

Work engagement is described as a positive, fulfilling work-related state of mind that is characterised by vigour, dedication and absorption [52]. Vigour refers to high levels of energy, mental resilience and the willingness to devote effort to one’s work even when faced with difficulties [57]. Dedication indicates that an employee is strongly involved in their work accompanied by a sense of meaningfulness, enthusiasm, inspiration and significance [56]. Absorption refers to being fully concentrated and happily absorbed in one’s work and finding it difficult to detach oneself from work [57]. Work engagement can predict organisational achievement, teamwork, and employee performance [58]. Future research on the topic is encouraged to consider employees’ specific contexts [58]. Despite the growth of research related to work engagement in nursing practice, the phenomenon has not been sufficiently studied [59], limiting opportunities to develop initiatives to improve work engagement [10]. Low work engagement can have detrimental effects on nurses and their outcomes; for example, Marti et al. [60] found a correlation between work engagement and burnout in a sample of Italian nurses. Managers were highlighted as imperative in implementing practices that improve work engagement and, consequently, healthcare outcomes [60]. A cross-sectional South African study by Patience et al. [28] also found that work engagement differed between public and private sector nurses, and this was generally influenced by meaningfulness, career advancements, leadership and management. A previous study also indicated that employees who experience work engagement may be more or less engaged on a specific day depending on the availability of job resources [61]. In addition, Breevart et al. [61] suggest that work engagement may not only vary on a specific day, but according to previous daily diary studies, work engagement also varies among different individual nurses [61]. Based on these arguments, it is reasonable to assume that variance in work engagement can be expected among different individual nurses on a daily basis. Even though existing research indicates that work engagement contributes to increased positive outcomes and decreased negative outcomes for nurses and healthcare organisations, respectively [10], a clear gap is evident in the literature when it comes to exploring the daily basic need satisfaction and work engagement of nurses in the public sector working nightshifts, also in the context of general perceptions of job demands and job resources.

### 1.5. Relationship between Need Satisfaction and Work Engagement

Need satisfaction has been found to significantly explain differences in work engagement [62,63]. These findings suggest that when nurses face job demands, they need a sense of fulfilment of autonomy, relatedness, and competence to be engaged at work [12]. The SDT suggests that if nurses’ working conditions cater to their need for autonomy and competence, they can be more engaged [11]. Therefore, according to Albrecht [12], it is essential to understand the degree of satisfaction for basic needs when dealing with and understanding nurses’ engagement in their working environments. Furthermore, Trépanier et al. [55] found that the satisfaction of nurses’ basic needs has been positively related to work engagement and negatively to burnout and turnover intention. Even though there is support for the relationship between daily need satisfaction and work engagement in previous studies, the dynamics of the satisfaction of nurses’ basic needs and the effect on work engagement in the public healthcare sector, in the context of job demands and job resources, is not clear. This investigation is especially imperative under the recent pressure on healthcare workers by the COVID-19 pandemic to work shifts and long hours [64].

To conclude, this study seeks to contribute to the literature on daily work engagement by investigating whether daily need satisfaction in the forms of autonomy, competence and relatedness influences variability of work engagement in nurses working nightshifts in the South African public sector. Hence, this study seeks to test the following hypotheses:

**Hypothesis** **1.** **(H1).** *Daily basic psychological need satisfaction, in the form of autonomy, significantly predicts variation in daily work engagement*.

**Hypothesis** **2.** **(H2).** *Daily basic psychological need satisfaction, in the form of competence, significantly predicts variation in daily work engagement*.

**Hypothesis** **3.** **(H3).** *Daily basic psychological need satisfaction, in the form of relatedness, significantly predicts variation in daily work engagement*.

Following this, any supported hypothesis will be further explored by isolating the variance of the specific need and then investigating the general job demands and job resources that may contribute to its variability. The same will be carried out for daily work engagement. This exploration will provide important insight from the general level variables.

## 2. Materials and Methods

### 2.1. Study Design and Population

For the purpose of this study, a quantitative, ‘shortitudinal’ design with a multi-level research approach was followed by means of which data were collected based on a daily diary survey method. The multi-level research is characterised by data that have a hierarchical or clustered structure, which takes into account both between-person and within-person variations [65]; thus, the variables of this study were measured to analyse and compare results. Nurses working nightshifts in a public hospital in the North West Province of South Africa had to complete daily paper-based questionnaires once a day after working nightshifts for seven consecutive workdays—in line with their nightshift cycle. The diary study approach captured the short-term dynamics of experiences in the work context by addressing questions concerning when employees felt engaged [66]. This procedure allowed for studying and examining of the predictors of change at both between and within levels [67]. Furthermore, at the start of the study, one-time questions were posed regarding the nurses’ general (overall) level of job demands and job resources (see the section on measuring instruments below for more information).

Table 1 presents a breakdown of the participants of the current study.

Purposive sampling [68] was implemented to include nightshift nurses in a public hospital in the North West Province. Nurses working in other hospitals in South Africa were therefore excluded, as permission was only available for this hospital. The sampled nurses completed informed consent forms and were reminded that their participation was voluntary and anonymous and that they may withdraw from the study at any time without any consequences. The data were gathered from 33 nurses on seven occasions (33 nurses × 7 occasions = 231; *n* = 231). The seven occasions were based on the seven-day shift cycle of the nurses, the first occasion being day one of the shift and the seventh occasion the seventh day of the shift. The average age of the participants was 41.30 years (SD = 9.94). The sample consisted of 26 (78.80%) female and 7 (21.20%) male nurses. In terms of ethnicity (labelled in line with the Employment Equity Act), 90.90% (*n* = 30) of participants identified as African employees, 6.10% were white employees (*n* = 2) and one participant was an employee of mixed ethnic origin. Furthermore, the majority (39.40%; *n* = 13) of the participants were professional nurses, whereas the rest of the participants consisted of 24.20% (*n* = 8) enrolled nurses, 21.20% (*n* = 7) nurse assistants and 9.10% (*n* = 3) auxiliary nurses, respectively. In terms of the departments in which the nurses were assigned to complete their nightshifts, the following was evident: the majority (27.30%; *n* = 9) of the participants worked in the surgical ward, 15.20% (*n* = 5) worked in the orthopaedic ward, 12.00% (*n* = 4) in the Intensive Care Unit and 9.10% (*n* = 3) of the participants worked in the medical, paediatric and premature wards, respectively.

### 2.2. Measures

The process and the measuring instruments were divided into two categories: (i) a general questionnaire in the form of a booklet with multiple questions were completed at the start of a shift to establish baseline information (68 items in total) and (ii) a shorter daily measuring questionnaire was used to discover the day-level information for the study (19 items in total). The shorter daily survey was considered to be appropriate and not too time-consuming.

*General work engagement* (α = 0.88) was measured with the 9-item Utrecht Work Engagement Scale [69], which has been validated in various contexts (e.g., Brazil [70]; Russia [71]). The 9-item Utrecht Work Engagement Scale has also been shown to be applicable within the South African context as a single latent factor due to high correlations between the individual components (e.g., [72]), which contains three vigour items (e.g., ‘At my work, I feel bursting with energy’), three dedication items (e.g., ‘I am enthusiastic about my job’) and three absorption items (e.g., ‘I get carried away when I am working’). Respondents were required to respond based on a seven-point frequency-rating scale, which ranges from (0) ‘Never’ to (6) ‘Always’. The Cronbach’s alpha coefficients vary between 0.85 and 0.92 [69].

*General need satisfaction* was measured by using the Work-Related Basic Need Satisfaction scale (WBNS) [73]. The scale was designed to measure the satisfaction of the needs for autonomy (α = 0.69; e.g., ‘I feel free to do my job the way I think it could best be done’), competence (α = 0.90; e.g., ‘I really master my tasks at my job’), and the need for relatedness (α = 0.62; e.g., ‘At work, I can talk with people about things that really matter to me’) [43]. The WBNS consists of 16 items which were scored on a five-point Likert scale which ranges from ‘Totally disagree’ (1) to ‘Totally agree’ (5). The Cronbach’s alpha coefficients were found to be 0.81, 0.85 and 0.82 on average for the three basic psychological needs: autonomy, competence, and relatedness [73].

*General job demands and general job resources* were measured with the Job Demands–Resources Scale (JDRS) developed by [74]. The JDRS consisted of 32 items which were scored on a four-point scale ranging from 1 (Never) to 4 (Always). The scales of the JDRS will be reliable with α > 0.70 within the South African context. Specifically, the following job demands and job resources were measured: work load (α = 0.72; e.g., ‘Do you have too much work to do?), emotional load (α = 0.61; e.g., ‘Does your work put you in emotionally upsetting situations?’), supervisor support (α = 0.60; e.g., ‘Can you count on your direct supervisor when you come across difficulties in your work?’), role clarity (α = 0.66; e.g., ‘Do you know exactly for what you are responsible?’), job information (α = 0.73; e.g., ‘Do you receive sufficient information on the results of your work?’), participation in decisions (α = 0.76; e.g., ‘Do you have direct influence on your department’s decisions?’), opportunities to learn (α = 0.87; e.g., ‘Does your job offer you the opportunities for personal growth and development?’), job autonomy (α = 0.83; e.g., Do you have freedom in carrying out your work activities?’), colleague support (α = 0.76; e.g., ‘Do you get on well with your colleagues?’) and remuneration (α = 0.94; e.g., ‘Do you think you are paid enough for the work that you do?’).

*Daily need satisfaction* was measured by means of a daily short questionnaire which consisted of the nine adapted items from the Work-Related Basic Need Satisfaction scale (WBNS) [75]. The three basic needs for autonomy, relatedness and competence were each measured by selected items. The wording of the items was changed slightly to make them applicable to day-to-day measurement (e.g., ‘Today, I felt free to do my job the way I think it could best be done’). Items were scored on a five-point Likert scale which ranges from ‘Strongly disagree’ (1) to ‘Strongly agree’ (5). The Cronbach’s alpha coefficients were found to be 0.81, 0.85 and 0.82 on average for the three basic needs: autonomy, competence and relatedness [73].

*Daily work engagement* was measured by applying the 9-item UWES. The items were included for each dimension and the wording of the items were changed to make them more applicable for the purpose of day-to-day measurement (e.g., ‘Today, I felt strong and vigorous’). Items were scored on a scale ranging from (1) ‘Strongly disagree’ to (5) ‘Strongly agree’. The Cronbach’s ranged from 0.85 to 0.90 [69].

### 2.3. Data Analysis

Data were investigated by employing Mplus 8.2 [76]. Descriptive statistics and correlations were also provided. Expressly, the means and standard deviations were provided and for the correlational relationships, the standard—small, medium and large—effect sizes were considered [77]. Specifically, multi-level analysis was used to test the hypotheses whereby repeated measures indicated variance both within participants over days and between participants. The intra-class correlation coefficient (ICC) was used to compare within- and between-person variance [78,79]. Therefore, the justification for using multi-level analysis would be sound if significant variation is indicated by the ICC. Specifically, a random intercept model and a random slope model were tested.

Given significant variation, as indicated by specific factors, a model(s) was utilised to ascertain the effect of general job demands and job resources on daily work engagement (within participants over days). Specifically, the variation in daily work engagement was isolated as a parameter in the model and the general job demands and job resources were regressed on this variation. For this model, Bayesian estimation was used, since specifying this type of random variance parameter is not available with maximum likelihood and Bayes is a powerful estimation method for smaller sample sizes—providing similar results when maximum likelihood would be feasible. For Bayesian modelling, the PSR convergence criterion was used which should be below 1.05 [79]. Furthermore, the parameter trace plots and kernel density plots were provided for any parameters of interest. The parameter trace plots should show sufficient mixing between the two chains and the kernel density plot should show a smoothed distribution for the parameters to be trusted for interpretation. The models were estimated with at least 50,000 iterations. The 95% credible intervals of the estimates were considered to ascertain the impact of the relationships. In terms of the functionality of adding priors to the Bayesian model, no priors were specified and the Mplus default priors were used as the default.

## 3. Results

For each of the daily variables the following ICCs were found: work engagement (53.8%), daily need satisfaction in the form of autonomy (25.2%), daily need satisfaction in the form of competence (40.3%) and daily need satisfaction in the form of relatedness (33.1%). Table 2 below reflects the descriptive statistics and correlations for these variables.

As depicted in Table 2, the mean values of all the scales were above the middle point of the five-point scale with similar standard deviations. As for the correlations, daily work engagement highly correlated with daily need satisfaction, both in the form of autonomy (*r* = 0.60; large effect) and competence (*r* = 0.67; large effect), but only had a small correlation with daily need satisfaction in the form of relatedness (*r* = 0.18; small effect). Daily need satisfaction of autonomy and competence were also highly related (*r* = 0.76; large effect), but both had low correlations with relatedness (*r* = 0.23, *r* = −0.10; small effects). We also calculated semi-partial correlations at a reviewer’s request based on the mean values of the daily means of the participant as there were some large correlational values present and none of these values were concerning (*r* < 0.62).

### 3.1. The Effects of Daily Basic Need Satisfaction on Daily Work Engagement as Outcome

Table 3 below reflects the results from the modelling of the effects of the three daily basic need satisfaction variables on daily work engagement—specifically for the intercept only and intercept and slope models.

The results indicated that daily need satisfaction in the form of autonomy (γ = 0.16; SE = 0.10; *p* = 0.11) and daily need satisfaction of relatedness (γ = 0.10; SE = 0.06; *p* = 0.09) did not significantly explain variation in daily work engagement. However, daily need satisfaction of competence indicated a significant increase over time in daily work engagement (γ = 0.34; SE = 0.09; *p* < 0.001). The AIC and SABIC statistics for the intercept and slope model were lower than the values for the intercept only model, even though this was not the case with the BIC—the difference was still below 10, which would have indicated a significantly improved model. Preference is also given to the SABIC as it also factors in the sample size. These results supported H2 and rejected H1 and H3 of the study. Consequently, the analyses entered an exploratory phase to investigate the effects of the general variables on the variability in daily need satisfaction of competence and daily work engagement.

### 3.2. The Impact of General Work Engagement, Job Demands–Resources on the Variability in Daily Need Satisfaction in the Form of Competence

**Figure 1:**
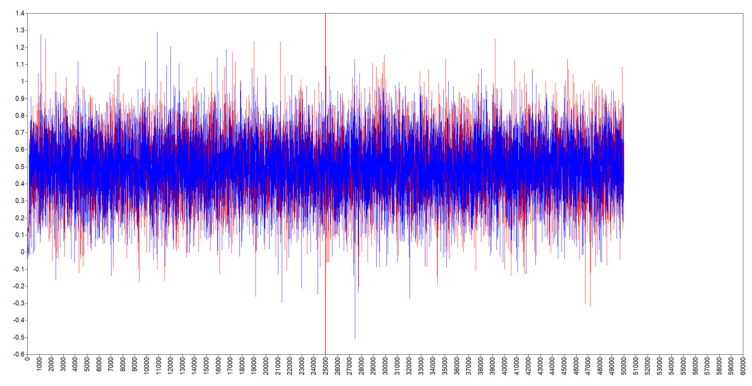
The chain-mixing trace plot for general emotional load on daily need satisfaction of competence.

**Figure 2:**
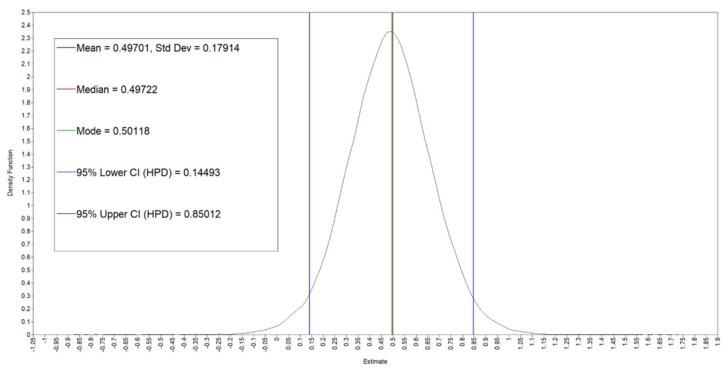
The kernel density plot for general emotional load on daily need satisfaction of competence.

Given the support for H2, this model explored the effect of the general-level variables measured at the start of the study on the variability of daily need satisfaction of competence. The model converged according to the PSR criteria of less than 1.05 within 300 iterations. However, to be more confident in the generated estimates, the iterations were increased to 50,000 to ensure a smoothed parameter distribution. Table 4 gives an account of the estimates from the model and the Figure 1 and Figure 2 present the trace plots and kernel density plots for the parameters of interest in the Bayesian model.

It is evident from Table 4 that general emotional load explained significant variability in daily need satisfaction of competence (γ = 0.50; SD = 0.18; 95% CI (0.15, 0.86)). It should be noted that the credible interval for general colleague support was close to not crossing zero (γ = 0.35; SD = 0.19; 95% CI (−0.02, 0.72)). None of the other variables were of interest, all crossing zero.

As can be seen the figures indicate sufficient chain-mixing and a normal distribution of the parameter. This was also the case for all other parameters in the model.

### 3.3. The Impact of General Work Engagement, Job Demands–Resources on the Variability of Daily Work Engagement

A model was also explored, including daily work engagement as the outcome and the job demands, job resources and general work engagement to gauge the impact of the general factors on daily work engagement. The model converged according to the PSR criteria of less than 1.05 within 5000 iterations. However, to be more confident in the generated estimates, the iterations were increased tenfold to 50,000 to ensure a smoothed parameter distribution. Table 5 gives an account of the estimates from the model and the figures present the trace plots and kernel density plots for the parameters of interest and for the Bayesian model.

Table 5 only indicates one variable that did not cross zero—general role clarity had a negative impact on the daily variability in work engagement (γ = −0.29; SD = 0.13; 95% CI (−0.53, 0.04)). Once again, general colleague support was close to crossing the threshold, but did not (γ = 0.18; SD = 0.10; 95% CI (−0.01, 0.37)). None of the other variables were of interest, all crossing zero by some margin.

## 4. Discussion

This study aimed to investigate the variation in nurses’ work engagement from day to day (7-day consecutive nightshifts) per the satisfaction of their three basic psychological needs as indicated by the SDT—autonomy, competence and relatedness—within a public healthcare context of South Africa. This study is the first multi-level diary study to consider all these factors in a single research study within public healthcare nursing. This research was vital as it contributes to the existing research that examines the relations between work-related basic need satisfaction and work engagement [80]. This study was also the first study to investigate the relationship between the satisfaction of nightshift nurses’ basic needs and the effect thereof on work engagement based on a multi-level diary study in the public healthcare sector of South Africa.

The results from the modelling of the effects of three daily basic need satisfaction variables on daily work engagement revealed that daily basic need satisfaction, in terms of autonomy, did not significantly predict variance in daily work engagement (H1 rejected). This result is contrary to the results of a recent cross-sectional study which revealed that the satisfaction of autonomy in the sense of control and freedom to exercise choice positively relates to work engagement within a South African agricultural business [81]. Autonomy also played a role in influencing nurses’ work engagement and resulting organisational behaviour in a study conducted in Wuhan, China [82]. Possible reasons why the current study contradicts these findings could be that professional autonomy in nursing is a complex issue, and the concept varies in comparison to other business sectors [83] and work environments. According to Christmals and Armstrong [84], there is a trend in placing lower cadre nurses in public Sub-Saharan health care facilities where much more autonomy is required, and low health outcomes are rampant. A study using an English sample of nurses indicated that their autonomy level depends on their own knowledge, competence, and confidence [85]. Education, legislation and organisational policy/culture may impact nurses’ perception of autonomy and their need to operate in a more professional independent manner [86]. For instance, professional autonomy for nurses means the ability to make decisions within their profession and their right and responsibility to act according to the standards of the nursing profession, for which they will be held accountable by the South African National Council [3,87]. Nurses’ work environments typically consist of receiving a minimal amount of time to perform autonomous tasks or make decisions, whereas the more significant amount of time is used for administering medications [88].

Daily basic need satisfaction, in the form of competence, significantly predicted variation in daily work engagement (H2 supported), indicating that competence explained significant variance in daily work engagement. This is in line with the findings of other studies indicating that the fulfilment of basic needs for competency positively influences work engagement [89]. Ghazawy et al. [90] also found that Egyptian nurses with higher education and certification had higher work engagement, whereas Athey et al. [85] reported that nurses who feel they fully utilise their skills had higher work engagement. Deci et al. [11] suggest that if nurses’ need for competence is fulfilled within their working conditions, they can be more engaged. Thus, the nurses’ daily working conditions, which cater to fulfilling the need for competence, may have varied on particular days, which caused variation in daily work engagement over time. The nurses might have been able to accomplish and master their assigned tasks on specific days, whereas the conditions might not have presented them with the opportunity on other working days. Modern nursing requires more decision making than in the past [3], and nightshift nurses often do not have access to specialists to assist in patient care decisions [6]. This nursing context highlights the importance of considering nurses’ need for competence and the importance of further analysing this hypothesis.

In terms of H3, results indicated that daily basic need satisfaction, in the form of relatedness, did not significantly predict variation in daily work engagement. Therefore, hypothesis 3 was not supported. This was contrary to expectations and the results of Vera et al. [57] and a similar study by [91], which demonstrated that the need for social support has a positive relationship with work engagement in nurses. These results indicate that even though the nurses might have experienced a mutual feeling of having supportive connections with colleagues [33] on a daily basis, it did not predict or have an impact on the variation in their daily level of work engagement. According to Van den Broeck et al. [42], the need for relatedness is occasionally characterised as being less important for some outcomes than the need for either autonomy or competence. Additionally, culture can also be a factor in determining the role of social support in nursing [92]. Thus, the need for relatedness might be less crucial for outcomes such as work engagement in this context and should be further explored. However, the results for general colleague support in the exploratory phase below should also be taken into consideration, which is discussed further down below.

In terms of the results from the modelling of the effects of three daily basic need satisfaction variables on daily work engagement, the results supported H2 and did not support H1 and H3 of the study. Consequently, the analyses further entered an exploratory phase to investigate the effects of the general variables (work engagement, job demands and job resources) on the variability in daily need satisfaction of competence and daily work engagement.

First, the effect of the general work engagement, general job demands and general job resources measured at the start of the study on the variability of daily need satisfaction of competence was explored. The results showed that general emotional load explained significant variability in daily need satisfaction of competence scores of the nurses. According to the JD-R model, emotional demands are job demands, and job demands contribute to burnout, especially in professions working with people daily, such as nursing [93]. The emotional demands of nursing refer to tasks such as paying attention, interpreting and understanding patients’ feelings and needs, and dealing with emotional situations such as dealing with relatives and restless patients [94]. Such emotional demands can cause nurses to experience overwhelming emotions, leading to negative consequences for their wellbeing and quality of patient care [32].

Furthermore, the high emotional demands that nurses face are a feature of the nursing profession and are evident throughout nurses’ daily working routines [5,95]. Therefore, emotional demands are interpreted by nurses as challenges that can provide opportunities to develop personal and professional abilities to cope with the changing demands of work [96]. How the participants interpreted their general emotional load in terms of being challenges or hindrances could have influenced their daily scores on competence. Nurses develop their competencies on dealing with emotionally stressful events by practising and observing, social support and guidance from other more experienced colleagues, psychological capital, job security, religion and routinisation of emotions [95,97]. This implies that colleagues’ support as a job resource plays an important role and has a positive effect, especially when nurses experience emotionally demanding conditions [32].

The credible interval for general colleague support was close to not crossing zero (γ = 0.35; SD = 0.19; 95% CI (−0.02, 0.72)); thus, it might be assumed that general colleague support may explain significant variability in daily NS: competence. Recent research has linked shift work and staffing levels to nursing competence [98]. This result can be explained in line with the findings of Kato et al. [99], indicating that colleague support plays a significant role in the satisfaction of autonomy, relatedness and competence needs. Furthermore, the findings of Di Muzio et al. [98] indicate that colleague support in terms of ‘lending a hand’ when dealing with stressful and demanding situations made nurses feel confident in their tasks. Feelings of competence allow nurses to be engaged and derive pride from their work [100]. Taking these findings into consideration, noting that nightshift nurses experience lower staffing levels, access to specialists and higher responsibility than day-shift nurses, these could be reasons for significant variability in daily need satisfaction of competence.

Next, the impact of general work engagement, general job demands and general job resources on the variability of daily work engagement was explored. The results indicated that general role clarity, a job resource, had a negative impact on the daily variability in work engagement (γ = −0.29; SD = 0.13; 95% CI (−0.53, 0.04)). Role clarity refers to the extent that an employee knows what is expected of them and what they need to do to achieve this expectation in their employment. This finding suggests that nurses who scored higher on role clarity showed less variability in daily work engagement scores—that is, if role clarity levels are high in general, and people know what is expected of them in their roles, their daily work engagement will be less variable. This finding is in line with the current literature, which states that job resources (e.g., role clarity) have been identified as the main drivers that predicts and contributes to work engagement over time as well as daily [101]. Role clarity is related to task performance; thus, employees should be informed on the standards they are expected to meet and be clear of what their supervisors or colleagues expect of them to improve their job performance [102]. Nursing role clarity is complex as it is influenced by a variety of factors such as legislation, regulatory frameworks, healthcare structures [103], nurse knowledge and population needs [104]. Hence, nursing management needs to master the task of clearly defining the roles of nurses based on the needs of patients and the knowledge required from the nurse to make effective decisions in daily practice [102]. Overall, nurses’ role clarity differs between shift types [104] and is a key factor in determining work engagement and impacts patient care and the health care team [105]. According to O’Rourke [106], promoting high-quality relationships between nurses and transformational supervisors or leaders leads to higher role clarity. Therefore, in general, if the participants of this study were familiar with and clear about what their supervisors or colleagues expected of them in their roles, it could have resulted in less variance in daily work engagement scores.

Once again, general colleague support was close to crossing the threshold, but did not (γ = 0.18; SD = 0.10; 95% CI (−0.01, 0.37)). Therefore, general colleague support might have an impact on the variability in daily work engagement. This is supported by Vera et al. [57] in an earlier multi-level study, which found that the greater the availability of job resources at the team level (e.g., colleague support), the more likely it is that nurses will be engaged. Furthermore, Yang et al. [106] state that, often, research focuses on the recipient of social support and often does not consider the influence thereof on the provider of social support, especially within demanding occupations such as nursing. Their findings suggest that providing colleague support could either positively or negatively impact the provider—especially negatively if the support is provided out of obligation [106]. Moreover, as mentioned previously within this section, working conditions in public hospitals in South Africa are less satisfactory in terms of the shortage of staff and the availability of job resources [107]. According to the findings of Jardien-Baboo et al. [8], nurses working in the South African public sector stated that limited resources served as a barrier to deliver expected patient-centred care. More specifically, nightshift nurses experience lower staffing levels than dayshift nurses [108]. Thus, the participants’ variability in daily work engagement might have been a result of changes in job resources in terms of access to colleague support on particular days or being either the provider or recipient of colleague support and the effects thereof could have had an influence. The current study assists in explaining the extent nurses’ work engagement varies from day to day in accordance with the degree to which the three basic psychological needs are satisfied in the daily work activity of nightshift nurses. Determining the influence of the three basic psychological needs on variance in daily engagement could make organisations aware of why employees display fluctuations in their performance daily. Organisations need to know that employees experience fluctuating levels of work engagement when performing their work because the variance in engagement levels influences employee, team and organisational outcomes [58].

Furthermore, to address colleague support, a supportive work environment and teamwork among nurses should be promoted—peer coaching programmes can be implemented during which colleagues can talk to and support each other through difficult times. To satisfy the need for competence, the effectiveness of the current organisation’s performance management systems could be reviewed. It should be determined whether management and employees understand the performance management system and what it entails. Continuous performance discussions should occur between supervisors and employees, which is an opportunity for supervisors to review an employee’s current progress against his/her performance management plan and to identify areas in which the employee can be supported. Therefore, the focus is on the employee and to assist them in performing at their best. Individual development plans should be created for each employee based on the outcomes of the performance discussion to identify the required training courses or interventions to address possible development areas. Job enrichment can also be considered to enhance competence levels among staff. With regard to role clarity, management should ensure each employee has a job profile, stipulating their required roles and responsibilities for the specific position. Legislation for the nursing profession, regulatory frameworks and healthcare structures need to be considered when compiling or updating job profiles [102].

The findings of this study are even more critical for healthcare organisations in the South African context, which face high turnover of public sector nursing due to adverse outcomes of workload, resources available, role uncertainty and poor working conditions [3,14,109]. Healthcare organisations need to consider strategies that can be implemented to eliminate fluctuations in the availability of job resources, especially role clarity, to ensure less variance in daily engagement.

Based on the results obtained in this study, the following possible interventions are proposed. Concerning addressing emotional demands, the need for enhanced training and support for nurses to enable them to manage the emotional demands throughout their daily routines has been recognised [110]. This study indicates the importance of implementing training programmes among nurses to provide them with tools to enable them to better cope with their daily emotional demands. The training needs to focus on enhancing nurses’ emotional competencies and self-awareness in terms of the trait—emotional intelligence. Emotional intelligence is considered an important work tool for nurses [111] which is vital to their resilience and mental health [112]. Nursing staff could be requested to complete an emotional intelligence questionnaire and the results could be used to understand staff’s emotional demands within their roles. The results would enable the organisation to overview staff’s suitability for the role and likelihood of success. Based on the results, development plans or formal coaching programmes can be proposed.

Some limitations of this study should be noted. The first limitation of the study was that it was only conducted within a public healthcare context of South Africa, which should be considered regarding the external validity of the results, i.e., generalisation. Furthermore, despite including all the nightshift nurses working in the chosen public hospital (33), the sample size remains somewhat small which should be considered when interpreting the results and future studies with larger samples are recommended. Due to the small sample size, we also considered a lower bound for reliability of 0.60 as acceptable. The study concentrated on nurses working nightshifts in the public health sector. Thus, for future research, additional investigation is necessary in other sectors and/or multiple organisations to determine whether the results can be compared and generalised beyond this professional group.

Secondly, the study was based on self-reports, which may raise questions on the measurement of bias [113]. However, evidence suggests that participants in diary studies appear to show minimal cognitive processing before reporting their current states during a certain point in time [114]. Furthermore, maintaining participation for seven (7) consecutive working days was challenging. As such, the researcher was committed to distributing and collecting questionnaires daily as promised to the participants. Hence, participants started trusting the process, which ensured continuous commitment and participation. Other implementations that could assist with this process could be mobile phone applications that can prompt nurses before or after each shift. However, the financial implications of this should be considered and how effective it might be compared to in-person booklets after shifts.

Lastly, we measured the job demands and job resources only on the general level, so we did not control for the daily variations of the job demands and job resources in our exploratory analyses. This might be an important avenue for future research, but finding a balance between survey length and maximum information will remain a challenge so as not to unnecessarily burden participants on each occasion of the study.

## 5. Conclusions

After considering the results, evidence has been presented that significant positive relationships were found between daily need satisfaction of competence and the variation in daily work engagement and for general emotional load’s impact on variability in daily competence, respectively. A negative relationship was also found between general colleague support and daily work engagement variability. Healthcare organisations need to address colleague support by promoting a supportive work environment and teamwork among staff; address emotional demands through interventions designed to increase staff’s level of self-awareness; and provide tools to help staff cope with their emotional demands. Healthcare organisations can explore competence levels among staff, job enrichment and effective performance management systems. These findings need to be addressed within healthcare organisations so as to manage daily variance in employee engagement levels, which will sustain performance and enable the achievement of organisational goals.

## Figures and Tables

**Table 1:** Characteristics of the participants (*n* = 33).

Item	Category	Frequency	Percentage (%)
Gender	Male	7	21.20
	Female	26	78.80
Ethnicity *	African	30	90.90
	Mixed Ethnic Origin	1	3.00
	White	2	6.10
Job Title	Professional Nurse	13	39.40
	Auxiliary Nurse	3	9.10
	Nurse Assistant	7	21.20
	Enrolled Nurse	8	24.20
	Other	2	6.10
Department	Orthopaedic	5	15.20
	Intensive Care Unit	4	12.00
	Premature	3	9.10
	Surgical	9	27.30
	Medical	3	9.10
	Paediatric	3	9.10
	Other	6	18.20

Note: * = Designations are used in line with the terminology of the Employment Equity Act, 55 of 1998.

**Table 2:** Descriptive statistics and correlation matrix for the day-level variables.

Variable	M	SD	1	2	3	4
1. Work engagement	3.30	0.66	1.00			
2. NS: Autonomy	3.69	0.60	0.60 ^b^	1.00		
3. NS: Competence	3.97	0.67	0.67 ^b^	0.76 ^b^	1.00	
4. NS: Relatedness	3.07	0.66	0.18	0.24	−0.10	1.00

Notes: NS = Need satisfaction; M = Mean; All correlations significant *p* < 0.05; ^b^ = Large effect.

**Table 3:** The results from the multi-level modelling.

	**Intercept Only**		**Intercept and Slope**	
**Variable**	**Est**	**SE**	**Est**	**SE**
Constant	1.15 ^†^	0.26	1.03 ^†^	0.39
NS: Autonomy	-	-	0.16 ^†^	0.10
NS: Competence	-	-	0.34 *^,†^	0.09
NS: Relatedness	-	-	0.10 ^†^	0.06
Log-likelihood				
AIC	302.99	-	298.41	-
BIC	323.65	-	329.39	-
SABIC	304.63	-	300.87	-
Variance	0.15 ^†^	0.04	0.01 ^†^	0.11

Note: SE = Standard error * = significant *p* < 0.05; ^†^ = unstandardized; AIC = Akaike Information Criterion; BIC = Bayesian Information Criterion; SABIC = Sample size-adjusted Bayesian Information Criterion.

**Table 4:** Estimates with 95% credible intervals.

Path	Estimate	Posterior SD	Lower 95%CI	Upper 95%CI
General work overload → Daily NS: Competence	0.08	0.16	−0.24	0.40
General emotional load → Daily NS: Competence	0.50 *	0.18	0.15	0.86
General supervisor support → Daily NS: Competence	−0.11	0.28	−0.66	0.46
General role clarity → Daily NS: Competence	−0.20	0.25	−0.67	0.30
General job information → Daily NS: Competence	0.22	0.25	−0.27	0.73
General Participation → Daily NS: Competence	0.52	0.32	−0.13	1.14
General opportunities to learn → Daily NS: Competence	−0.60	0.32	−1.21	0.03
General job autonomy → Daily NS: Competence	−0.01	0.20	−0.41	0.37
General colleague support → Daily NS: Competence	0.35	0.19	−0.02	0.72
General remuneration → Daily NS: Competence	0.12	0.18	−0.22	0.47
General work engagement → Daily NS: Competence	−0.04	0.04	−0.13	0.05

Note: * = Did not cross zero; SD = standard deviation; CI = credible interval; NS = need satisfaction.

**Table 5:** Estimates with 95% credible intervals.

Path	Estimate	Posterior SD	Lower 95%CI	Upper 95%CI
General work overload → Daily work engagement	−0.01	0.08	−0.18	0.15
General emotional load → Daily work engagement	0.07	0.09	−0.11	0.25
General supervisor support → Daily work engagement	0.08	0.14	−0.19	0.37
General role clarity → Daily work engagement	−0.29 *	0.13	−0.53	−0.04
General job information → Daily work engagement	−0.11	0.13	−0.36	0.14
General participation → Daily work engagement	0.06	0.16	−0.27	0.37
General opportunities to learn → Daily work engagement	0.00	0.17	−0.32	0.33
General job autonomy → Daily work engagement	−0.05	0.10	−0.25	0.16
General colleague support → Daily work engagement	0.18	0.10	−0.01	0.37
General remuneration → Daily work engagement	0.09	0.09	−0.09	0.27
General work engagement → Daily work engagement	−0.01	0.02	−0.05	0.04

Note: * = Did not cross zero; SD = standard deviation; CI = credible interval.

## Data Availability

The associated analyses and data for the current study can be requested from the corresponding author. All reasonable requests will be considered.
